# Localization Reliability Improvement Using Deep Gaussian Process Regression Model

**DOI:** 10.3390/s18124164

**Published:** 2018-11-27

**Authors:** Fei Teng, Wenyuan Tao, Chung-Ming Own

**Affiliations:** College of Intelligence and Computing, Tianjin University, Tianjin 300350, China; 2016218046@tju.edu.cn (F.T.); taowenyuan@tju.edu.cn (W.T.)

**Keywords:** location estimation, RSS fingerprinting, deep gaussian process, reinforcement learning

## Abstract

With the widespread use of the Global Positioning System, indoor positioning technology has attracted increasing attention. Many systems with distinct deployment costs and positioning accuracies have been developed over the past decade for indoor positioning. The method that is based on received signal strength (RSS) is the most widely used. However, manually measuring RSS signal values to build a fingerprint database is costly and time-consuming, and it is impractical in a dynamic environment with a large positioning area. In this study, we propose an indoor positioning system that is based on the deep Gaussian process regression (DGPR) model. This model is a nonparametric model and it only needs to measure part of the reference points, thus reducing the time and cost required for data collection. The model converts the RSS values into four types of characterizing values as input data and then predicts the position coordinates using DGPR. Finally, after reinforcement learning, the position coordinates are optimized. The authors conducted several experiments on a simulated environment by MATLAB and physical environments at Tianjin University. The experiments examined different environments, different kernels, and positioning accuracy. The results showed that the proposed method could not only retain the positioning accuracy, but also save the computation time that is required for location estimation.

## 1. Introduction

With the continual development of information technology and smart phones worldwide, the Global Positioning System (GPS) is being used increasingly in daily life. Although GPS can accurately and reliably locate signals in indoor environments, it cannot penetrate thick walls and cannot be used effectively indoors. Therefore, indoor positioning requires further study.

In recent years, many indoor positioning technologies have been proposed, such as Radio Frequency, Infrared, Ultrasound, Optical, and Magnetic Field Strength with the relevant positioning calculation mechanism [1]. The main types of indoor positioning algorithmic mechanisms are triangulation location and fingerprint positioning. Received signal strength (RSS)-based location fingerprinting is one of the most popular methods in indoor positioning [2]. RSS location fingerprints are a unique set of RSS signal values for each location. An RSS fingerprinting database is initially created for mapping the relationships between each physical location and its unique RSS fingerprint. The RSS fingerprint of the device location is matched to the information in the RSS fingerprint database, and then the location of the most similar entry to the sample is chosen as the estimated location [3]. This method has two phases: offline training phase and online position estimation phase. The offline training phase measures the positioning environment, collects signal strength values of each position in the target positioning area, and finally saves the collected data to establish a fingerprint database. The online position estimation stage matches the received real-time signal strength values with the fingerprint database established in the offline stage during positioning [4].

The main problem in RSS-based fingerprint recognition is that RSS values of any location can be affected easily by factors, such as multiple paths, shadow effects, and crowd interference. This means that RSS values that are measured at the same location at different times may show differences [4,5]. The RSS fingerprint that was obtained at different times may not match the previous fingerprint stored in the database, leading to incorrect estimation results. In other words, the data in the fingerprint database is actually static data, and the real-time positioning value is dynamic data. When we measure RSS fingerprint samples at the test position, the localization algorithm may match an incorrect estimated location from the static database [3,5]. Although the matching fingerprint data appears to be the most likely data in the fingerprint database, the corresponding physical location of the fingerprint may be very far from the physical location corresponding to the correct fingerprint data. Therefore, before repositioning, finding the fingerprints of each position in the entire positioning area once again enables very accurate positioning. However, doing so is costly and time-consuming when the positioning area is very large. In particular, it is impractical to continually update the fingerprint database in an area where the interference continues to change [6].

Gaussian process regression (GPR) can solve the abovementioned problem of high costs in offline measurement data. In this approach, the calibration effort and costs of building the RSS signature map are reduced by using the model signal strength that was obtained using an advanced Gaussian process (GP), and the problems faced with the RSS signature map are solved in the online phase [7]. GP is a nonparametric model that is completely characterized by its average function and covariance matrix [8]. Some studies have used a GP to generate a fingerprint database in the offline phase [7]. Farid et al. (2013) demonstrated how to use a GP to generate likelihoods at locations for which no calibration data were available, and they proved that Gaussian regression could be applied successfully to various localization problems [9]. Fernando Seco et al. (2010) proposed the use of an observation model that is based on GP nonparametric regression to represent the RSS distribution of the specific environment of each RFID tag [10]. Aravecchia et al. (2014) proposed the use of GP to interpolate the signal vector received in the offline stage to estimate the device position during the test [11]. These studies have proved that GPR is a viable means of improving positioning accuracy and reducing the cost of collecting fingerprinting data. Furthermore, Bisio et al. (2017) defined two-folded methods to solve the location problem. First, a Gaussian Process (GP) is used during the training (offline) phase of an indoor positioning algorithm to generate the fingerprint database. Furthermore, during the positioning (online) phase, and a smart algorithm used for reducing the computation effort for positioning calculation [12]. On the contrary, when considering indoor dynamic surveying data for this purpose, the Gaussian process regression based radio-map construction method exploiting both realistic and virtual indoor dynamic surveying data is proposed and evaluated in [13]. The results show that this method can obtain not only high accuracy but also high availability in the indoor service area, even though the realistic indoor surveying data is rare. Seco et al. (2010) present a Bayesian method for an indoor RFID location system that uses an observation Gaussian processes nonparametric regression model to represent the environment-specific RSS distributions for the individual RFID tags [10].

Yiu et al. (2015) used GPR for RSS indication (RSSI) prediction to solve indoor location problems [14]. First, they took partial measurements from the area of interest. They then used the Firefly algorithm to train and categorize the prior results derived using GPR. The trained results were used to build a refined fingerprinting database for the entire area of interest. The experiment showed that the GPR model could achieve a satisfactory result in predicting the RSS of an area with no prior measures. However, according to [10], the ability to expand the GPR model is restricted by the need to rebuild the fingerprinting database; in addition, the flexibility in the computation of the GPR models was limited in their study [7]. In [7], Liu et al. (2016) improved the GPR system and proposed the GPRP system. In GPRP, the GP prior distribution was used for regression and for predicting the RSS at locations with no prior measurements, and the naive Bayes algorithm was used to derive the obtained conditional Gaussian probability. It overcame previous shortcomings and it precluded the need to establish a fingerprint database. When compared with Yiu’s method, the calculation was simplified, thereby saving time and cost, and the positioning results were more accurate. However, in the prediction, the values of position information *x* and *y* need to be calculated separately (the probability interval of both *x* and *y* needs to be found, and then, naive Bayes is used to select the most possible position information in the interval), and GPRP cannot directly draw (*x,y*) as the positioning process is cumbersome. Therefore, to directly obtain the *x* and *y* values and further improve the positioning accuracy, in our study, the authors used a model that was based on the deep Gaussian process regression (DGPR) model to perform positioning through mobile phone on both simulated and physical environments.

The remainder of this manuscript is organized as follows. Section 2 reviews related studies on the properties of RSSI for signal transmission and defines GP and DGP. Section 3 describes the DGPR measurement system used in the current study and describes the data analysis method. Section 4 discusses the experiments that were performed in the simulated and real environments. Finally, Section 5 presents the conclusions of this study and provides recommendations for future research.

## 2. Preliminaries

### 2.1. RSS Fingerprint

The core concept of RSS-based location fingerprinting is that each location has a unique set of signal values, and when the system starts up, the mobile device receives the system’s unique RSS value. Then, the device searches the fingerprint database and identifies the most similar value to the system-unique RSS value of the estimated position [15]. When compared with traditional triangulation techniques, the RSS fingerprinting method does not necessitate knowledge of the location of the base station before measurement, and it is unnecessary to accurately synchronize and establish the propagation model of the indoor environment. In addition, RSS fingerprinting has very high accuracy, and therefore, it is widely used in various indoor positioning systems. Currently, two problems affect indoor positioning that are based on RSS signals. (1) The fingerprint database established at each location in the target positioning area is acquired during the offline training phase, and this database needs to be updated constantly over time with changes in the environment. (2) The positioning accuracy varies with the dynamic environment [6]. As mentioned, RSS is vulnerable to issues, such as multiple paths, shadow effects, and people’s actions. As the location environment changes, radio signals transmitted from APs to the mobile devices are affected to varying degrees, and the positioning accuracy decreases.

During signal propagation, as the propagation distance increases and environmental interference occurs, the signal strength weakens and attenuates. The wall attenuation factor (WAF) model can describe the slow fading and attenuation of RSS signals in the indoor environment. The WAF is used to predict the signal propagation when the wall is the main obstacle [6]. The formula for this model is as follows:

(1) $$p\left(d\right)=p\left(d_{0}\right)-10\times n\times \log\left(\frac{d}{d_{0}}\right)-\{\begin{array}{c} NW\times WAF\;NW<C\\ C\times WAF\;NW\geq C \end{array}$$

Here, *n* represents the signal attenuation rate as the distance increases; *d* is the distance between the base station AP and the signal transmitting end, $p\left(d_{0}\right)$ is the signal strength at reference distance $d_{0}$, *WAF* is the wall attenuation factor, and *NW* is the number of walls between the measurement point and the base station. The constant *C* is the number of wall tolerances that the attenuation factor affects the signal’s worth. In other words, when *NW* is greater than or equal to *C*, the attenuation factor no longer affects the signal strength. The signal value of the measurement point is obtained using the WAF model, and the position corresponding to the RSS subset that matches the value that is most similar to the value is retrieved in the fingerprint database as the estimated position. The K-nearest neighbor algorithm is the most commonly used matching method. We can use the simplest Euclidean distance to determine the location of the nearest neighbor. The Euclidean distance is calculated, as follows:

(2) $$Eucdist\left(S,R\right)=\sqrt{\sum_{i=1}^{n}{\left(s-r_{i}\right)}^{2}}.$$

Here, *S* is the RSS value of the measurement location and *R*, the RSS value closest to *S* in the fingerprint database, *n* denotes the number of data.

### 2.2. Gaussian Process Regression

The GPR is a new machine-learning method that is based on Bayesian theory and statistical learning theory. It provides a flexible framework for probabilistic regression, and it is widely used to solve high-dimension, small-sample, or nonlinear regression problems [7]. GP is a set of joint Gaussian distributions for any finite number of random variables [16]. The GP estimates Gaussian distributions over functions based on training data [17], the results can be seen as linear combinations over kernel functions centered on the training observations, which highlights the capability of GP to model arbitrary smooth functions. The GP is fully specified by a mean function and a covariance function, such as

(3) $$\{\begin{array}{c} m\left(x\right)=E\left[f\left(x\right)\right]\\ k\left(x|x^{\prime }\right)=E\left[\left(f\left(x\right)-m\left(x\right)\right)\left(f\left(x^{\prime }\right)-m\left(x^{\prime }\right)\right)\right] \end{array}$$

where m(x) is the mean function, $k\left(x|x^{\prime }\right)$ is the covariance function, *x* and $x^{\prime }\in R$ are random variables. The GP equation is $f\left(x\right)\sim gp\left(m\left(x\right),\;k\left(x,x^{\prime }\right)\right)$. For simplification, the mean function is usually set to zero in the data preprocessing stage [7].

The GP aims to model the following noisy process [11]:

(4) y=f(x)+ε

where $x\in R^{d}$ is the input vector; f(x) is the function value; and, y is the observed value of f(x) affected by noise *ε*, and $\varepsilon \sim N\left(0,\sigma^{2}\right)$.

According to the formula of GP, the prior distribution of observed values y is

(5) $$y\sim N\left(0,K\left(X,X\right)+\sigma_{n}^{2}I_{n}\right)$$

where K(X,X) is a n×n symmetric positive definite covariance matrix, and $I_{n}$ is an n-dimensional unit matrix. Because the key assumption in GP modeling is that the data can be represented as a sample from a multivariate Gaussian distribution, we can infer the joint prior distribution of observation y and prediction $f_{*}$, as follows:

(6) $$\left[\begin{array}{c} y\\ f_{*} \end{array}\right]\sim N\left(0,\left[\begin{array}{cc} K+\sigma_{n}^{2}I_{n} & K_{*}^{T}\\ K_{*} & k_{**} \end{array}\right]\right)$$

where K denotes the covariance function, $K_{*}=Kx_{*},X$ is covariance matrix between the test point $x_{*}$ and the input X of the training set, *T* indicates matrix transposition, K=K(X,X) and $k_{**}=k\left(x_{*},x_{*}\right)$ are the covariance matrix of test point itself. Then, we can calculate the posterior distribution of the forecast value $f_{*}$ as

(7) $$P(f_{*}|X,y,x_{*})\sim N\left({\bar{f}}_{*},cov\left(f_{*}\right)\right)$$

where

(8) $${\bar{f}}_{*}=k_{*}{\left[K+\sigma_{n}^{2}I_{n}\right]}^{-1}y$$

(9) $$\mathrm{cov}\left(f_{*}\right)=k_{**}-k_{*}\times {\left[K+\sigma_{n}^{2}I_{n}\right]}^{-1}K_{*}^{T}$$

Note that ${\hat{\mu }}_{*}={\bar{f}}_{*}$ and ${\hat{\sigma }}_{f_{*}}^{2}=\mathrm{cov}\left(f_{*}\right)$ are the mean and variance of the prediction value $f_{*}$, respectively [16].

### 2.3. Gaussian Process Regression

The DGP is a deep network in which each layer uses the GP for modeling; it enables a deep probabilistic nonparametric approach to flexibly tackle complex machine-learning problems with sound quantification of uncertainty [18]. DGPs are multilayer hierarchical generalizations of GPs and they are formally equivalent to neural networks with multiple, infinitely wide hidden layers [19]. DGPs retain useful properties of GPs, such as nonparametric modeling power and well-calibrated predictive uncertainty estimates [19], it can also overcome the limitations of single-layer GPs that can only represent a restricted class of functions [20]. DGPs are richer models than standard GPs, just as deep networks are richer than generalized linear models. In contrast to models with highly parameterized kernels, DGPs learn a representation hierarchy nonparametrically with very few hyperparameters to optimize [21]. The deep model seems to have structural advantages that can improve the quality of learning in complicated data sets that are associated with abstract information [22].

**Figure 1:**
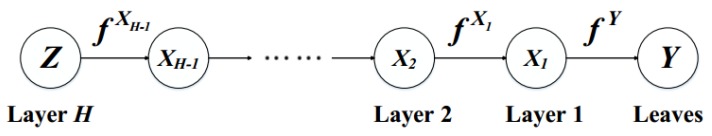
Deep Gaussian Process architecture.

The DGP architecture corresponds to a graphical model with three types of nodes, as shown in Figure 1, leaf nodes *Y*, parent latent node *Z*, and intermediate latent spaces. The nodes at each level are both the output of the previous layer and the input of the next layer. Leaf node *Y* is the measurement point, and $Y\in R^{N\times D}$. The intermediate latent node is $X_{h}\in R^{N\times Q_{h}}$, where h=1, 2,⋯, H−1, and *H* is the number of hidden layers and the parent latent node is $Z=X_{H}\in R^{N\times Q_{z}}$.

A DGP is generated, as follows:

(10) $$\begin{array}{c} Y_{nd}=f_{d}^{Y}\left(X_{1}\right)+\varepsilon_{nd}^{Y},d=1,\cdots ,D\\ \vdots \\ X_{{H-1}_{nq}}=f_{q}^{X_{H-1}}\left(Z_{n}\right)+\varepsilon_{nd}^{X_{H-1}},q=1,\cdots ,Q,Z_{n}\in R^{Q_{z}} \end{array}$$

where ε is Gaussian with variance $\sigma_{\epsilon }^{2}$, d and q are the numbers of intermediate layers of the deep model, $D\;and\;Q\in N^{*}$, $f_{q}^{X_{H-1}}\left(.\right)$ is the GP function of X in *H*-1th layer, and $X_{{H-1}_{nq}}$ is the observed value of data $x_{n}$ in the *H*-1th layer. As mentioned, the intermediate node is involved in two GPs, playing the role of an input and an output, respectively. As shown in Figure 1, node $X_{1}$ participates in the two GPs of input $f^{X_{1}}\sim gp\left(0,K^{X_{1}}\right)$ and output $f^{Y}\sim gp\left(0,K^{Y}\right)$. According to the solution process of GPR, the posterior distribution of a DGP is

(11) $$p\left(Y\right)=\int_{X,Z}p\left(Y|X_{1}\right)p(X_{1}|X_{2})\cdots p\left(X_{H-1}|Z\right)p\left(Z\right)$$

Suppose that we have a training set comprising *N* D-dimensional inputs $x_{n}$ and observation pair $\left(x_{n},y_{n}\right)$. The probabilistic representation of a DGP comprising *L* layers is [19]

(12) $$p\left(f_{l}|\theta_{l}\right)=gp\left(f_{l};0,K_{l}\right),l=1,\cdots ,L$$

(13) $$p\left(h_{l}|f_{l},h_{l-1},\sigma_{l}^{2}\right)=\underset{n}{\prod }N\left(h_{l,n};f_{l}\left(h_{l-1,n}\right),\sigma_{l}^{2}\right),\;h_{l,n}=x_{n},\;h_{L,n}=y_{n}$$

(14) $$p\left(y|f_{L},h_{L-1},\sigma_{L}^{2}\right)=\underset{n}{\prod }N\left(y_{n};f_{L}\left(h_{L-1,n}\right),\sigma_{L}^{2}\right)$$

where $h_{l,n}$ is a hidden layer and $f_{l}$, the function of each hidden layer.

## 3. System Design

### 3.1. System Overview

**Figure 2:**
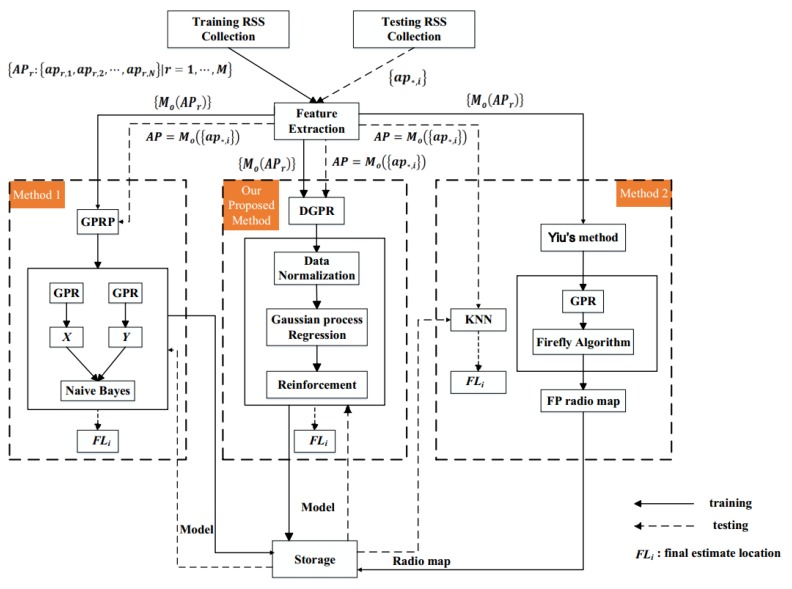
Comparison of frameworks of Deep Gaussian Process Regression Model (DGPR) method and methods.

Yiu et al. used a trained GPR model to estimate the signature map of an area [14]. For optimizing hyperparameters in GPR, they proposed that the signature map should be rebuilt; however, inefficient computations restricted the flexibility of the Gaussian model. Thus, Liu et al. proposed the GPRP system for solving this problem [7]. Although the fingerprint database reconstruction problem was solved, the positioning process is complicated and the position coordinates *(x,y)* cannot be obtained directly. Therefore, in our study, we used DGPR to reduce the complexity of the indoor positioning system. While maintaining the flexibility of the GP model, we could also overcome the limitations of single-layer GP and further improve the positioning accuracy. Figure 2 shows the proposed system framework and then compares it with those of Yiu et al. method and the Liu et al. method.

All three methods were used to create a discrete and partial RSS fingerprinting database in the testing area. Accordingly, we derived and tested several GP models for system computation. The different GP models had distinct distribution functions and hyperparameters. However, Yiu et al. proposed a method that is based on categorized and GP algorithms and finished with the rebuilding of the RSS fingerprinting database [14]. As described in the previous section, recreating the fingerprint database is costly and time-consuming. The GPRP method that was proposed by Liu et al. used the naive Bayes method to select all predicted locations from the GP models; thus, the computations were simplified and made less time-consuming. However, as shown in Figure 2, the method that was proposed by Liu et al. cannot directly obtain the target position through GPR [7]. Each regression result does not provide a specific value; instead, it provides a probability region. Then, the target location is selected based on naive Bayes. To further simplify the positioning process and improve the positioning accuracy, we propose the DGPR model. In Figure 2, the solid line indicates the training phase and the dashed line indicates the testing phase. $FL_{i}$ was derived as the final estimated location for each method. In this figure, we tried to illustrate the fewer computation steps that are needed in our method.

### 3.2. The Improved Deep Gaussian Process Regression Model (DGPR)

**Figure 3:**
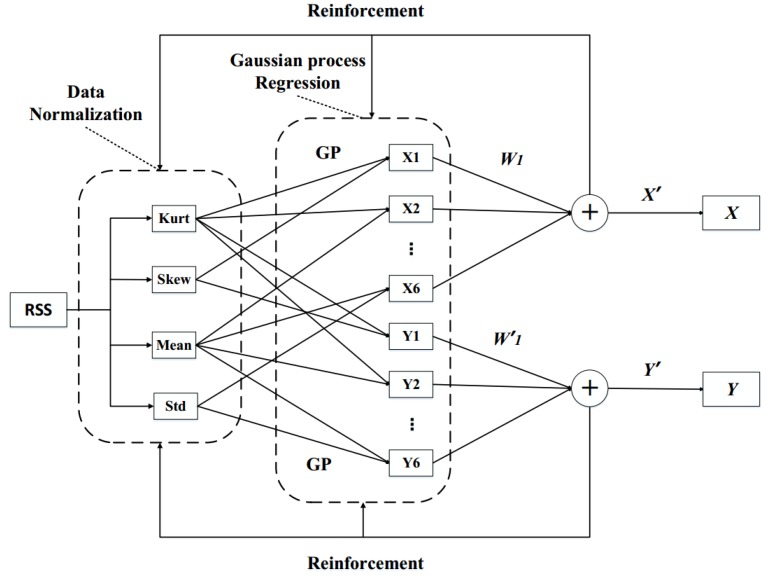
Deep Gaussian Process Regression model.

The DGPR model is divided into three parts, as shown in Figure 3, data normalization, Gaussian process regression, and reinforcement.

In our method, first, an offline RSS fingerprinting database must be built. We assume that the testing area has *M* reference points in locations $L_{r}\left(r\in \left\{1,2,\cdots ,M\right\}\right)$. In the sampling phase, we collect the RSS value at the rth reference point of *N* APs from 1 to j times, that is (*N* is the number of APs),

(15) $$AP_{r}=\left\{ap_{r,1},ap_{r,2},\cdots ,ap_{r,N}\right\}$$

where

(16) $$ap_{r,h}=M_{o}\left(\overset{j}{\underset{i=1}{\sum }}ap_{r,h}^{i}\right)$$

$M_{o}\left(.\right)$ is the mod function used to derive the remainder of the division. In Equation (16), this function computes the multiple RSS signal values that were received from the base station *h* at the *r* location into one value, and the divisor is j, which is the number of RSS that measured at the position *r*, and the dividend is the sum of RSS signal strength values. According to our experiences, because of the influence of multipath of the signal, the mod function can get rid of the unstable values than the other computation methods [7]. $ap_{r,N}^{i}$ represents the RSS signal value that was measured by the *j*-th time of the *N*-th AP at the *r*-th reference point (*N* is the number of APs); $ap_{r,1}$ denotes the signal strength of the first base station received at the *r-th* reference point; by contrast, $AP_{r}$ is the filtering set of signal strength received at the rth reference point.

When we attempt to predict the unknown position at the input of RSS values, we obtain a series of RSS values $AP_{*}$, as follows:

(17) $$AP_{*}=\left\{ap_{*,1},ap_{*,2},\cdots ,ap_{*,N}\right\}$$

Because of the noises in our environment, we can coincide with the regression problem as $L\left(AP_{*}\right)=f\left(AP_{*}\right)+v$ for simplification. The latent function f(.) describes the relationship between the RSS and spatial coordinates. v denotes the physical noise; this value follows a Gaussian distribution. Thus far, we have obtained the input data of the model $AP_{*}$. Next, the input data $AP_{*}$ is provided to the DGPR model that we have proposed for positioning.

#### 3.2.1. Data Normalization

The first layer in the DGPR model is data normalization, and this model mainly uses four characterizing values of the signal for regression prediction: kurtosis (kurt), skewness (skew), mean, and standard deviation (std). These characterizing values are described herein. The positioning data based on the RSS positioning method is single, mainly because this method only provides RSS values. To add more different types of data while improving the positioning accuracy, we extracted multiple features from the collected RSS data and used RSS feature values as data sources [23].

1. Mean is the average value, where N is the number of APs, $ap_{*,i}$ is used to represent the value of RSS in the *i*-th APs. Thus, the mean RSS value for all the APs, $\bar{AP_{*}}$ is listed, as follows,
  (18) $$\bar{AP_{*}}=\frac{1}{N}\overset{N}{\underset{i=1}{\sum }}ap_{*,i},$$
2. Standard deviation is derived from RSS data $AP_{*}$.
  (19) $$std=\sqrt{\frac{1}{N}\overset{N}{\underset{i=1}{\sum }}{\left(ap_{*,i}-mean\right)}^{2}},\;n=1,2,\cdots ,N$$
3. Kurt is used to describe the peak-to-peak characterizing values of the probability density distribution curve at averaging. It is expressed as
  (20) $$k=\frac{\frac{1}{N}\sum_{i=1}^{N}{\left(ap_{*,i}-\bar{AP_{*}}\right)}^{4}}{{\left(\frac{1}{N}\sum_{i=1}^{N}{\left(ap_{*,i}-\bar{AP_{*}}\right)}^{2}\right)}^{2}}-3$$
  where $ap_{*,i}$ is the RSS value measured by the *i*-th AP at the measurement position. $\bar{AP_{*}}$ is the mean value that is derived from Equation (18).
4. Skew is used to measure the asymmetry of the probability distribution. It can be positive, negative, or zero. Positive and negative values, respectively, indicate that a value is on the right- or left-hand side of the probability density function; a zero value indicates that values are distributed relatively evenly on both sides of the mean. The skew is calculated as
  (21) $$s=\frac{E\left[AP_{*}{}^{3}\right]-3\bar{AP_{*}}\sigma^{2}-{\bar{AP_{*}}}^{3}}{\sigma^{3}}$$
  where $\bar{AP_{*}}$ is the center distance derived from Equation (18), $AP_{*}$ is the RSS signal strength value measured at the anchor point, σ is the standard deviation in $AP_{*}$. Because $AP_{*}{}^{3}$ is the cubic value of this RSS signal strength value, hence, we derived $AP_{*}{}^{3}$ as ${\bar{AP_{*}}}^{3}$, and $E\left[AP_{*}{}^{3}\right]$ is computed as the expected value in our study.

**Figure 4:**
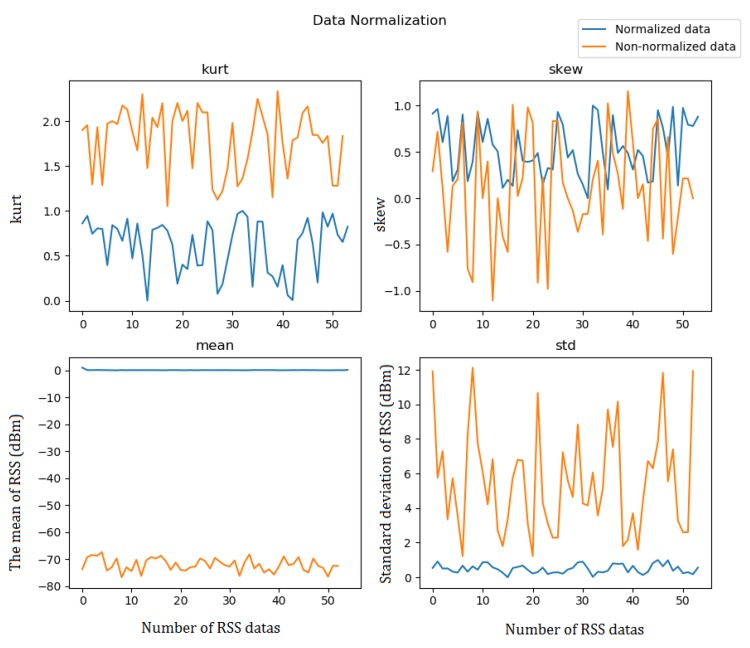
Normalized results of four characterizing values.

To make the data processing more convenient and to improve location accuracy, we need to normalize these four characterizing values and map the data to the [0,1] range for processing. Data normalization removes the unit limits of the data and converts data into a dimensionless pure value, thereby facilitating the comparison and weighting of indicators of different units or magnitudes, that is, turning dimensioned expressions into dimensionless expressions. The units of the signal value’s four characterizing values are different. Data normalization is necessary and it is beneficial for calculating the data and improving the model accuracy. Data can be normalized in many ways. This study uses the Z-score standardization method:

(22) $$z=\frac{x-m}{\sigma }$$

where *x* is the input data for data normalization; *m*, the mean; and, σ, the standard deviation. Figure 4 shows the normalized results of the four data characterizing values, namely, kurtosis, skewness, mean, and standard deviation.

#### 3.2.2. Gaussian Process Regression

The GPR is the core of the DGPR model. Normalized data that is calculated by the previous layer is used as input data for the GPR. The four characterizing values that were obtained are intersected one by one and used as the parameters of the GPR to predict the regression, which can directly predict the specific position coordinates of the location point. In the previous section, after the four feature values were normalized, the $AP_{*}$ value was expressed as

(23) $$AP_{*}\to \{\begin{array}{c} K_{*}\left(k_{*,1},k_{*,2},\cdots ,k_{*,N}\right)\\ Sk_{*}\left(sk_{*,1},sk_{*,2},\cdots ,sk_{*,N}\right)\\ M_{*}\left(m_{*,1},m_{*,2},\cdots ,m_{*,N}\right)\\ St_{*}\left(st_{*,1},st_{*,2},\cdots ,st_{*,N}\right) \end{array}$$

where $K_{*}$ is the kurtosis eigenvalue; $Sk_{*}$, the skewness eigenvalue; $M_{*}$, the average eigenvalue; and, $St_{*}$, the variance eigenvalue. These four groups of data are used as the input data of GPR. Because of the noise in our environment, we can combine the regression problem with it as follows:

(24) $$\left(x,y\right)=f\left(fe_{n1},fe_{n2}\right)+N\left(0,\sigma_{n}^{2}\right),$$

where $fe_{n1}$ and $fe_{n2}$ are characterizing values, n1,n2∈{1,⋯,4}, that is, $fe_{n1}$ and $fe_{n2}$ correspond to $fe_{n1},fe_{n2}\in \left\{kurt,skew,mean,std\right\}$, and n1≠n2; $N\left(0,\sigma_{n}^{2}\right)$ is the physical noise that follows a Gaussian distribution; (*x,y*) is the resulting positional coordinate; and, f(·) is a potential function that describes the relationship between these four characterizing values and spatial coordinates,

(25) $$f\left(fe_{n1},fe_{n2}\right)\;\sim \;GP\left(m\left(fe_{n1},fe_{n2}\right),k\left(\left(fe_{n1},fe_{n1^{\prime }}\right),\left(fe_{n2},fe_{n2^{\prime }}\right)\right)\right),$$

where $fe_{n1^{\prime }}$ is the characterizing value calculated using the RSS data set ($AP_{r}$) collected in the offline stage. According to the solution of GPR, the posterior distribution is

(26) $$p\left(\left(x,y\right)|f,\sigma_{n}^{2}\right)=\underset{n}{\prod }N\left((x_{n},y_{n}\right);f\left(fe_{n1},fe_{n2}\right),\sigma_{n}^{2}).$$

GPR is defined by the mean and covariance function. The mean and covariance function represent the main features and structure of GPR. Many types of mean and covariance functions are commonly used in GPR. Tables, Table 1 and Table 2 respectively list the commonly used mean and covariance functions. The GPR performance differs with the mean and covariance kernel functions used. In Section 4, we experimentally tested the effects of different covariance functions on GPR performance. In this study, zero and constant kernel were respectively used as the mean and covariance function.

#### 3.2.3. Reinforcement

In the previous layer, the input data consists of multiple sets of two characterizing values. The GPR algorithm is used to predict a set of positional coordinates *(X,Y)* of the positioning point as $\left(X,Y\right)=\left\{\left(x_{1},y_{1}\right),\left(x_{2},y_{2}\right),\cdots \left(x_{s},y_{s}\right)\right\},\;s=1,2\cdots ,6$. To locate the coordinates more accurately, we use reinforcement/enhancement learning to make the model proceed from the existing state and to constantly optimize our own strategy to obtain the position coordinates $\left(x_{*},y_{*}\right)$ with the smallest error.

At first, the reinforcement learning is mainly a decision-making process, and the goal of reinforcement learning is to enable machines to learn a good strategy independently to solve some decision-making problems. Q-learning algorithm is the most typical reinforcement learning algorithm [24,25]. Q-learning is a model-independent reinforcement learning algorithm that was proposed by Watkins based on time difference by means of state-action logarithmic function Q(s,a). It is also called off-policy TD [25]. This study uses the concept of deep *Q*-learning (deep reinforcement learning), and the deep GP model that we proposed uses a network of *Q*-values. The *Q*-values is the action evaluation function $Q^{*}\left(s,a\right)$, which is used to indicate the potential value of a state. Starting from the state s, the cumulative reward of the strategy * is executed after the action a is performed. The parameter θ is the weight of each network layer in the model. The objective is to find the θ value that can obtain the minimum positional error by constantly updating the state and action. In this process, we need to constantly update the valuation function $Q^{*}\left(s,a;\theta \right)$. This is expressed as

(27) $$Q^{*}\left(s,a;\theta \right)=Q\left(s,a;\theta \right)+\alpha \left(r+\gamma max_{a^{\prime }}Q\left(s^{\prime },a^{\prime };\theta \right)-Q\left(s,a;\theta \right)\right)$$

where $Q^{*}\left(s,a;\theta \right)$ is the value function that represents the future potential value of a state and that needs to be updated; a, the action; s, the state; $Q\left(s^{\prime },a^{\prime },\theta \right)$, the value function under the optimal decision; and, $Q^{*}\left(s,a;\theta \right)$, the updated valuation function. Updating the value function actually implies updating the parameter θ. We need to update the parameter θ to make the Q(s,a;θ) function approximate the optimal $Q\left(s^{\prime },a^{\prime },\theta \right)$ value. Therefore, a loss function is defined to evaluate the difference between the predicted values and the target values:

(28) $$L\left(\theta \right)=E\left[{\left(TargetQ-Q\left(s,a;\theta \right)\right)}^{2}\right]$$

where the objective function TargetQ is

(29) $$TargetQ=r+\gamma max_{a^{\prime }}Q\left(s^{\prime },a^{\prime };\theta \right)$$

Although the target Q is calculated based on the value iteration, it does not directly assign this Q estimate value directly to the new Q. Instead, it uses a gradual approach in a manner that is similar to a gradient descent and approaches the target in small steps. Finally, it can converge to the optimal Q value. The gradient of L(θ) is calculated and the stochastic gradient descent method is used to update the parameter θ:

(30) $$\theta_{j}=\theta_{j}+\alpha \left(Q{\left(s,a;\theta \right)}^{\left(i\right)}-TargetQ^{\left(i\right)}\right)Q_{j}^{\left(i\right)}$$

where α is the learning rate/step size. After calculating θ values, the position coordinates $\left(x_{*},y_{*}\right)$ can be calculated as

(31) $$\left(x_{*},y_{*}\right)=\left(\frac{\sum_{n=1}^{M}\theta_{n}x_{n}}{M},\frac{\sum_{n=1}^{M}\theta_{n}y_{n}}{M}\right),M=1,2,\cdots 6.$$

## 4. Experiments and Discussion

### 4.1. Experiment Environment Initialization

**Figure 5:**
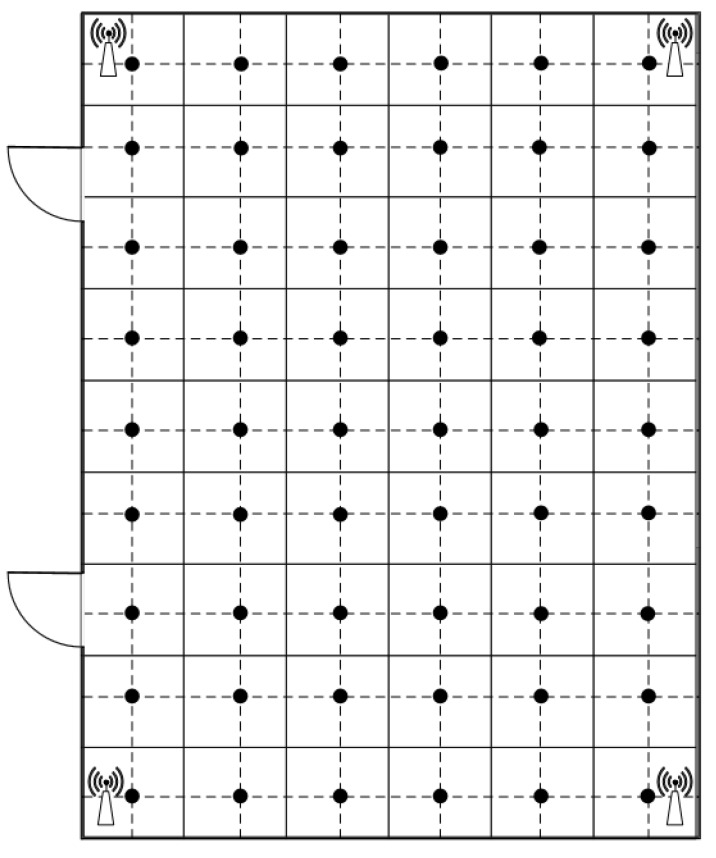
Physical testing area.

We conducted experiments in two environments. One environment was a simulation programmed using MATLAB R2017b. For the purpose of demonstrateing the flexibility of our proposed method, we focused on the comparison of two extreme sizes of the testing areas. In the simulation experiment, which was a 30 m×30 m square area with four beacons that were deployed at the corners. The signal was smooth; thus, the RSS energy declined gradually from the source, and the received energy could be predicted easily according to the distance from the source. In this area, the performance of the proposed model can be reviewed clearly. Furthermore, the physical experiment did perform on the second floor in the 55th office building of Tianjin University. This area was used to verify the accuracy and effectiveness of our positioning method in Section 4.2 and Section 4.3. The area was approximately 7 m×15 m (Figure 5). The area was a typical building hall with some pillars in each corner of the area and was relatively open in the center, with people being able to walk around freely. Four Bluetooth transmitters were placed in the corners to ensure full signal coverage in the testing area. Although this area is smaller than the simulated area, the real process of signal transmission can be revealed too. To receive the online RSS data, the Samsung cell phone (Note 3) was used as the data collector in the testing areas, according to our previous studies, the antenna design of Samsung cell phones was much more stable than the other brands at the data collection [7]. According to the broadcasting performance of Bluetooth beacon, we setup our transmitter up to 100 packages per second and 20 samples are collected at each location for the system evaluation [26,27]. Fifteen packages and five left pages were used for the system training and testing, respectively.

The simulated area and physical testing area were divided into 800 and 54 locations for the measurements, respectively. The locations in physical area are marked by black dots in Figure 5. The collected samples are transmitted to the server, the positioning coordinates are calculated by different methods in our experiments, and then the position information is returned to the mobile phone. In addition, we applied our DGPR method with the mod function to avoid potential overfitting problems through a single signal measurement.

### 4.2. Effect on Different GPR Kernel

In this study, we tested the following five kernel functions, Rational Quadratic Kernel, Constant Kernel, Matern Kernel, White Kernel, and Exp-Sine-Squared Kernel in our proposed Different GPR Kernel. All of the differences among these kernels were discussed on Section 3.2.2. The main use-case of White Kernel is as part of a sum-kernel, where it explains the noise component of a signal. Tuning its parameters corresponds to estimating the noise level. The Matern Kernel is a generalization of RBF and an absolute exponential kernel that is parameterized by an additional parameter *nu*. Rational Quadratic Kernel can be seen as a scale mixture (an infinite sum) of RBF kernels with different characteristic length scales. Exp-Sine-Squared Kernel allows for the modeling of periodic functions. It is parameterized by a length-scale parameter *length_scale* > *0* and a periodicity parameter *periodicity* > *0*. Constant Kernel can be used as part of a product-kernel where it scales the magnitude of the other factor (kernel) or as part of a sum-kernel where it modifies the mean of the GP.

**Figure 6:**
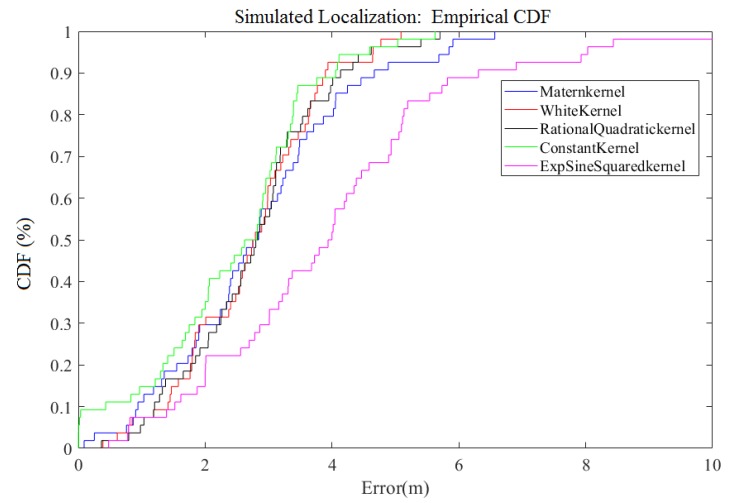
The CDF Comparison for Simulated localization error of different kernels.

Table 2 shows the expressions of these five kernels. We compared the results of the five kernels; the definition of each kernel is illustrated on Table 1 and Table 2. Table 3 summarizes the overall results. Obviously, the result of the Exp-Sine-Squared Kernel is the worst and the Constant Kernel works best in the DGPR model. On the other hand, Figure 6 shows the cumulative distribution function (CDF) plot of the final positioning errors for these five kernels. It is obvious that the positioning using the Constant Kernel is the most accurate. Therefore, we use the Constant Kernel as the kernel of the GPR in the DGPR middle layer.

### 4.3. Localization Accuracy in Different Environments

In these experiments, we evaluated our system performance in two different environments: simulated and field. In the field experiment environment, the location examined was the second floor of the 55th building of Tianjin University. We compared four methods for indoor localization, our proposed method, GPRP proposed by Liu et al., the method proposed by Yiu et al., and the general FingerPrint (FP) method. FP is based on the RSS location system and it requires the RSS mapping database of an entire area to be built. When comparing to the other methods, the offline database construction time and online indexing time cost more than the other three methods. Besides, in this experiment, we focused on the user experience on the location estimation in our study, the construction time of the offline database was ignored herein. On the contrary, the time consumption was briefly summarized by the data collection time and model execution time.

We used the test on the field environment as an example. The area was divided into 54 blocks. Each block was approximately 1.4 m long and 1.2 m wide. For every time, collecting the FP RSS of each block cost us 10 s, totally it took us about 54×10=540 s for the collection of 54 blocks, and the FP method took another 1 s to obtain/index the online location. Furthermore, in the methods that were proposed by Yiu et al. [14], Liu et al. (GPRP) [7], and us (DGPR), 30% sampling blocks were selected to collect their databases. Thus, the base RSS data collection time required 54×0.3×10=160 s. Overall, the model online execution times are 147 s, 45 s, and 28 s for Yiu et al., Liu et al. (GPRP), and us (DGPR), respectively. Then, the total time consumptions are 541 s, 307 s, 205 s, and 188 s by the same methods’ order. Table 4 shows the data for these four methods. These results indicate that the computation time increases with the sampling size of the FP database, because the procedure of RSS value needed to recollect every time. The less sampling time and fast model execution time would improve the users’ experiment. Comparing to the GPRP method, our proposed DGPR method can derive the position (*x, y*) directly without the inference method needed; the model execution time can be outperformed by the others. Thus, our proposed method can not only save the computation time that is required for collecting the RSS database, but also decrease the execution time on the system execution.

**Figure 7:**
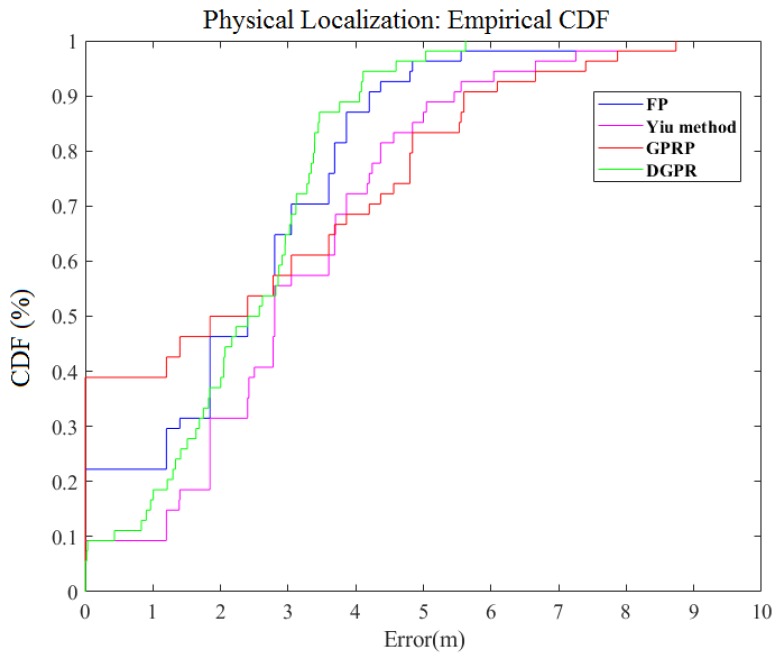
Comparison of CDF for physical localization error of different algorithms.

Furthermore, we compared four methods for the FP method, the method that was proposed by Yiu et al. [14], the GPRP method [7], and our proposed DGPR method. Figure 7 shows a comparison of these methods. The average errors for these respective methods were 2.36 m, 3.09 m, 2.34 m, and 2.28 m, respectively. In the meantime, if we reviewed the results from Figure 7, our method clearly reduces the maximum positioning error; our DGPR method’s maximum error is approximately 6 m. This error value is approximately 3 m, 3 m, and 2 m lower than that the Liu’s method, Yiu’s method, and FP method, respectively. Their probabilities of the maximum error of these three methods exceeding 6 m are 20%, 10%, and 2%, respectively. These results indicate that our proposed method can retain accuracy and reduce the computation time in the localization system.

**Figure 8:**
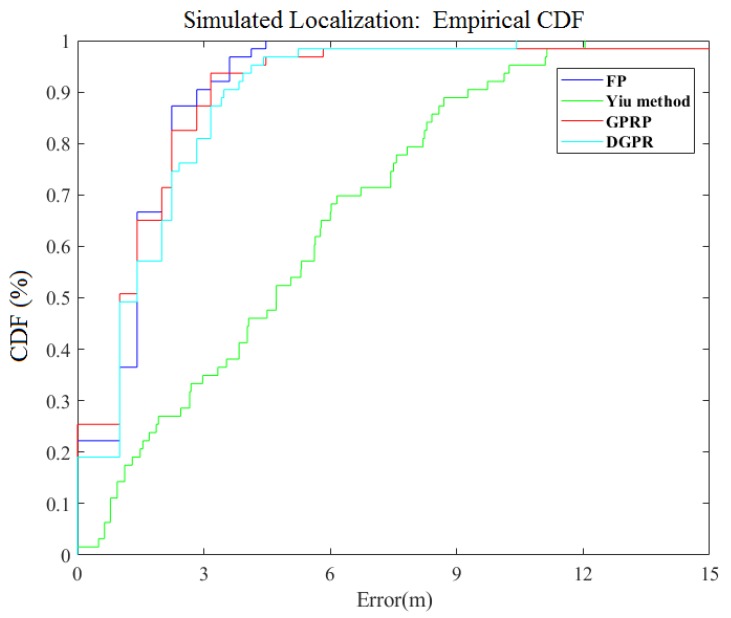
Comparison of CDF for simulated localization error of different algorithms.

In the simulation area, as shown in Figure 8, the results showed that the performance of our method was almost the same as that of the FP and GPRP methods, the average error of these three methods was 1.78 m, 1.87 m, and 1.46 m. The FP positioning method is relatively more accurate, but it takes the longest time; specifically, it is almost three times slower than our method. Although the method that was proposed by Liu et al. takes almost the same time as ours, it is slower for both actual measurements and simulation predictions. The maximum error of the method that was proposed by Liu et al. is extremely large, and it differs from ours by approximately 5 m. The method proposed by Yiu et al. takes less time than FP; however, it has the worst positioning accuracy with error of 4.88 m. Overall, the current results indicate that our proposed method can retain accuracy and reduce the computation time in the simulated localization system.

### 4.4. The Robustness for Location Accuracy Testing

To check the effects of obstacles on signal transmission in these simulations, we tested signal transmission in two situations: one used the same environment with different obstacles and the other situation involved a robust test with different obstacles.

**Figure 9:**
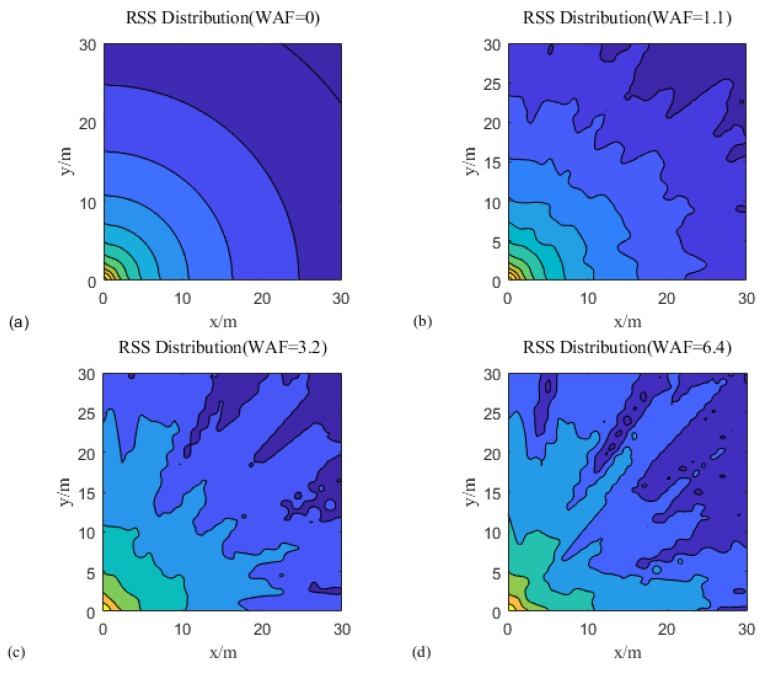
Received signal strength (RSS) strength mapping with wall attenuation factor (WAF) obstacles.

According to Equation (1), the WAF is defined as the obstacle factor, which is used to represent the effects of obstacles on the RSS. Figure 9a shows the optimal situation with receiving signals; the distribution is shown as a cyclic shape. In addition, Figure 9b–d show the strength mapping, with WAF of 1.1, 3.2, and 6.4, respectively. These values were indicated as light, medium, and heavy crowds in the environment. These figures show that the WAF affects the simulation environment; the larger the WAF, the greater is the signal interference, and the more obvious is the image fluctuation.

**Figure 10:**
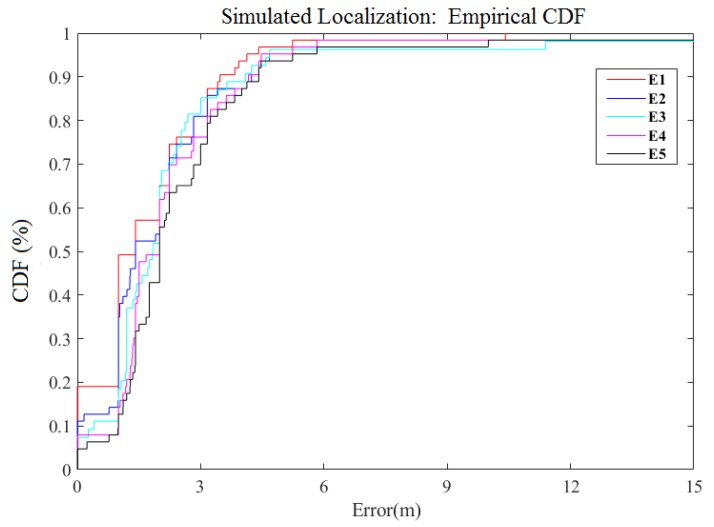
CDF of simulated localization error for different obstacle environments.

To test the robustness, the validation was performed according to the changing environment. Thus, the WAF value was changed in offline training and online testing. We defined four different environments in Table 5. These four environments were represented by the same offline training and dynamic online testing with light, medium, heavy, and super heavy crowds. E1 is the same situation for both offline training and online forecasting. It also emulates the experimental environment that is discussed herein. E1 is used for comparisons with other different environments to verify the robustness of our proposed DGPR model. Table 6 shows that, as the WAF increases, the interference of the environment also increases. Although the positioning accuracy has been decreased slightly, the overall difference is not great. Table 6 and Figure 10 show that the DGPR method could adjust to dynamic environments with acceptable accuracy. According to these examples, we can see that the positioning accuracy is nearly unchanged in both light and heavy crowds.

## 5. Conclusions

Generally, the main challenge in RSS-based location positioning is the high sensitivity of the technique to environmental changes. Variations in RSS measurement reduce estimation accuracy. In other words, if the radio propagation signal strength were correlated with the distance between the transmitter and the receiver, then location determination would be a trivial problem. However, the relationship between these two parameters is dynamic rather than straightforward. Therefore, many methods have been proposed for obtaining location predictions when the receiver position is changing. One such basic method is using an FP database, which involves straight computations for RSS signal collection. However, time consumption and searching from the lookup table database are both bottlenecks in this method. In this study, we proposed an indoor positioning system that is based on the DGPR model. This method can improve both the computation time and the efficiency.

In our study, we tested our method by examining the 7 m×15 m area in the 55th building of Tianjin University as a physical environment, and simulated environment with the 30 m×30 m. square area by MATLAB. We tested the performances on the different GPR Kernels, and derived the accuracy and time consumption with four distinct methods, including the general FingerPrint method, Yiu method in [14], GPRP method in [7], and our proposed DGRP method. Finally, the robustness for location accuracy was evaluated. Depending on our testing results, the constant kernel is the most accurate on the first position test. We adopt the constant kernel as our major part in our system. Then, we compare the localization accuracy with four different methods. The results shown that our proposed method can not only save the computation time required for building the RSS FP database, but also retain the accuracy of online prediction with the average error 1.46 m, 4.88 m, 1.87 m, and 1.78 m, respectively, for the simulated environment, and 2.34 m, 3.09 m, 2.28 m, and 2.30 m, respectively, for the field environment. Furthermore, on the robustness comparison, the validation was performed according to the changing environment with light, medium, heavy, and super heavy crowds’ areas. According to the results, we can see that the positioning accuracy is nearly unchanged in both light and heavy crowds. Thus, the robustness can be regarded as keeping the nearest accuracy in our proposed method.

## Figures and Tables

**Table 1:** Summary of several commonly used mean functions.

Mean Function	Equation Expression
Zero	0
Constant	$C;{\left(x\times x^{\prime }+\sigma_{0}^{2}\right)}^{p}$
Linear	αx+b
Poly	$\sum_{i}^{N}\alpha^{i}x^{N-i}$

**Table 2:** Summary of several commonly used covariance functions.

Covariance Function	Equation Expression
Rational Quadratic Kernel	$k\left(x,y\right)=1-\frac{\|x-y{\|}^{2}}{\|x-y{\|}^{2}+c}$
Exp-Sine-Squared Kernel	$k\left(x,y\right)=exp\left(-2sin{\left(\frac{pi/(per\times d\left(x,y\right)}{l}\right)}^{2}\right)$
White kernel	$k\left(x,y\right)=k_{1}\left(x,y\right)+k_{2}\left(x,y\right)$
Matern Kernel	$\begin{array}{l} k\left(x,y\right)\\ =\frac{1}{2^{v-1}\gamma \left(v\right)}{\left(\frac{2\sqrt{v}\|x-y\|}{\theta }\right)}^{v}H_{v}\left(\frac{2\sqrt{v}\|x-y\|}{\theta }\right) \end{array}$
Constant Kernel	$k\left(x,y\right)=\sigma_{0}^{2}$

**Table 3:** Localization results in testing area.

Kernel	RMSE (m)	1 m	2 m	3 m	6 m
Rational Quadratic Kernel	2.75	5.56%	24.04%	57.41%	100%
Exp-Sine-Squared Kernel	4.05	7.41%	16.67%	29.63%	88.89%
White Kernel	2.71	7.43%	29.63%	62.97%	100%
Matern Kernel	2.83	11.11%	29.64%	57.43%	98.15%
Constant Kernel	2.44	14.81%	33.33%	64.81	100%

**Table 4:** Total performance of four comparison methods.

	Simulated Experiments		Field Experiments	
	Time Cost (s)	Average Error (m)	Time Cost (s)	Average Error (m)
FingerPrint (FP) Method	4201	1.46	541	2.34
Yiu Method [14]	1385	4.88	307	3.09
GPRP Method [7]	1395	1.87	205	2.28
DGPR (Our Proposed Method)	1316	1.78	188	2.30

**Table 5:** Robustness testing for five different environments.

ID for Testing Environment	WAF for Off-Line Training	WAF for On-Line Testing
E1 (General area)	0.2	0.2
E2 (Light Crowds Area)	0.2	0.3
E3 (Medium Crowds Area)	0.2	0.5
E4 (Heavy Crowds Area)	0.2	1
E5 (Super Heavy Crowds Area)	0.2	2

**Table 6:** Localization results in different obstacle environments: E1 (General Crowds Area), E2 (Light Crowds Area), E3 (Medium Crowds Area), E4 (Heavy Crowds Area), E5 (Super Heavy Crowds Area).

	RMSE (m)	1 m	3 m	5 m	9 m
E1	1.78	49.21%	79.37%	95.24%	98.41%
E2	2.25	34.92%	79.37%	93.65%	96.83%
E3	2.26	14.81%	81.48%	96.29%	96.29%
E4	2.46	14.29%	74.60%	95.16%	98.39%
E5	2.72	11.11%	73.02%	92.06%	96.77%
